# Optimal Duration of Daily Antituberculosis Therapy before Switching to DOTS Intermittent Therapy to Reduce Mortality in HIV Infected Patients: A Duration-Response Analysis Using Restricted Cubic Splines

**DOI:** 10.1155/2014/704980

**Published:** 2014-12-15

**Authors:** Gerardo Alvarez-Uria, Raghavakalyan Pakam, Manoranjan Midde, Praveen Kumar Naik

**Affiliations:** Department of Infectious Diseases, Rural Development Trust Hospital, Kadiri Road, Bathalapalli, Anantapur, Andhra Pradesh 515661, India

## Abstract

Compared with thrice-weekly intermittent antituberculosis therapy (ATT), the use of daily ATT during the intensive phase has shown improved survival in HIV infected patients with tuberculosis. However, the optimal duration of daily ATT before initiating intermittent ATT is not well known. In this study, we analysed the mortality of HIV-related tuberculosis according to the duration of daily ATT before switching to thrice-weekly ATT in patients who completed at least two months of treatment in an HIV cohort study. Statistical analysis was performed using Cox proportional hazard models. To relax the linearity assumption in regression models and to allow for a flexible interpretation of the relationship between duration of daily ATT and mortality, continuous variables were modelled using restricted cubic splines. The study included 520 HIV infected patients with tuberculosis and 8,724.3 person-months of follow-up. The multivariable analysis showed that the mortality risk was inversely correlated with the duration of daily ATT before switching to intermittent therapy during the first 30 days of ATT but, after approximately 30 days of treatment, differences were not statistically significant. The results of this study suggest that daily ATT should be given for at least 30 days before switching to intermittent ATT in HIV infected patients with tuberculosis.

## 1. Introduction 

Approximately 13% of the 8.7 million incident cases of tuberculosis and 30% of the 1.4 million deaths from tuberculosis occur in people living with HIV [1]. In low- and middle-income countries, tuberculosis is the leading cause of mortality among HIV infected patients [2].

With an incidence of 2.3 million cases, one out of every four cases of tuberculosis worldwide occurs in India [3]. The Indian National Tuberculosis Programme follows a standardized thrice-weekly direct observed treatment short course (DOTS) strategy [4]. However, the current available evidence indicates that, compared with thrice-weekly ATT, the use of daily antituberculosis therapy (ATT) is associated with lower risk of treatment failure and mortality in HIV infected patients [5, 6].

To the best of our knowledge, the optimal duration of daily ATT before switching to intermittent ATT to reduce the mortality of HIV-related tuberculosis has not been formally studied. The aim of this study was to investigate the relationship between the mortality and the duration of daily ATT in HIV infected patients with tuberculosis in an HIV cohort study.

## 2. Methods

The study was performed in Anantapur, Andhra Pradesh, India. In Anantapur, 72% of the population lives in rural areas [7], and the HIV epidemic is characterized by being largely driven by heterosexual transmission, low CD4 cell counts at presentation, poor socioeconomic conditions, and high levels of illiteracy [8–10]. Rural Development Trust (RDT) is a nongovernmental organization that provides medical care to HIV infected people free of charge.

The Vicente Ferrer HIV Cohort Study (VFHCS) is an open cohort study of all HIV infected patients who have attended RDT hospitals. The cohort is fairly representative of the HIV population in the district, as it covers approximately 70% of all HIV infected people registered in the district [11]. The baseline characteristics of the cohort have been described in detail elsewhere [8].

For this study, we selected HIV infected adults (>15 years) from Anantapur District, not previously treated for tuberculosis, who started ATT from January 1, 2011, to February 20, 2013, and completed at least two months of ATT. In patients having several episodes of tuberculosis during the study period, only the first episode of tuberculosis was taken into account. Patients with tuberculous meningitis were excluded, as they received an intensified ATT with higher penetration in cerebrospinal fluid [12]. The selection of patients from the database was executed on February 14, 2014 (end of the follow-up period).

During the study period, antiretroviral therapy (ART) was freely available in the district. Patients diagnosed with tuberculosis were started on daily ATT. Daily ATT was given through fixed-dose combinations (two tablets of rifampicin 225 mg, isoniazid 150 mg, pyrazinamide 750 mg, and ethambutol 400 mg). At any time, patients could opt to receive thrice-weekly ATT under the Indian National Tuberculosis Programme, which provides antituberculosis drugs through a decentralized network of primary healthcare facilities. The Indian National Tuberculosis Programme follows the standard thrice-weekly DOTS strategy during six months for patients with no previous history of ATT [4]. Rifampicin, isoniazid, pyrazinamide, and ethambutol were given for two months (intensive phase), followed by rifampicin and isoniazid for four months (continuation phase) [13]. The duration of daily ATT before initiating intermittent ATT was calculated using the electronic records of the hospital pharmacy.

Multivariable survival analysis was performed using Cox proportional hazard models. Time was measured from 60 days after ATT initiation to death. Patients who did not die during the study period were censored at their latest visit date. To relax the linearity assumption in regression models and to allow for a flexible interpretation of the relationship between continuous covariates and mortality, continuous variables were transformed using restricted cubic splines with four knots [14]. The selection of covariates included in the multivariable analysis was made based on the results of previous studies from our cohort investigating prognostic factors in HIV-related tuberculosis [15, 16].

The statistical analysis was performed using Stata Statistical Software (Stata Corporation, Release 12.1, College Station, TX, USA). The VFHCS was approved by the ethical committee of the RDT Hospital.

## 3. Results

**Figure 1:**
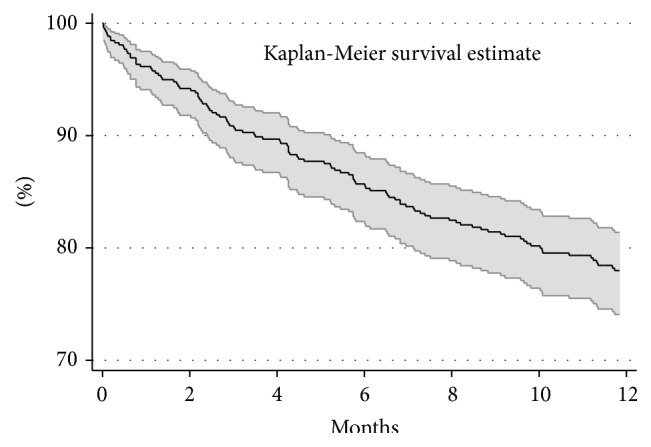
Survival curve and 95% confidence interval of HIV infected patients with tuberculosis after two months of antituberculosis therapy in Anantapur, India.

The study included 520 HIV infected patients with tuberculosis and 8,724.3 person-months of follow-up. The survival estimate with 95% confidence interval is presented in Figure 1. The estimated survival at 12 months was 78% (95% confidence interval [CI], 74.1–81.4).

Baseline characteristics are shown in Table 1. Over one-third were women, 2.9% were homeless, over half were illiterate, 21.2% had sputum positive smear, and 5.2% had disseminated tuberculosis. Over one-fourth of patients were on ART at the time of ATT initiation, and the majority of those who were not on ART started ART within two months of ATT.

**Figure 2:**
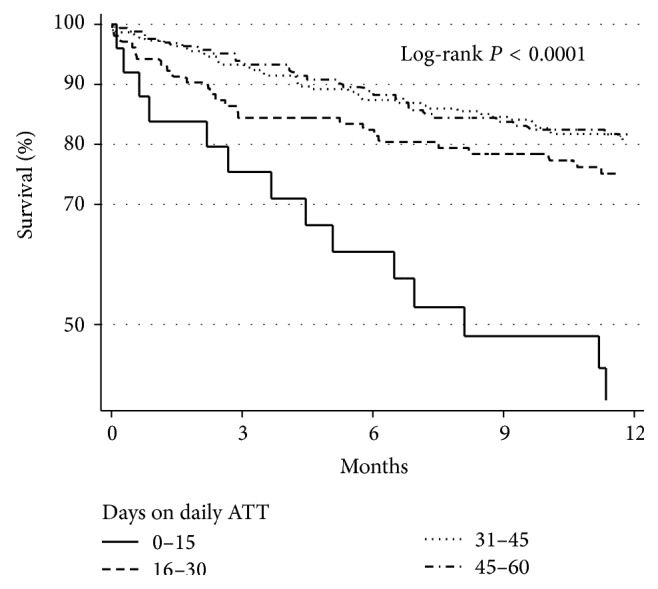
Kaplan-Meier survival estimates of HIV infected patients with tuberculosis grouped by duration of daily antituberculosis therapy (ATT) before switching to thrice-weekly ATT during the first two months of treatment.

Kaplan-Meier survival estimates by number of days on daily ATT are presented in Figure 2. The group that received 1–15 days of daily ATT had the highest mortality, followed by those who received 16–30 days of daily ATT. However, the mortality of those who received 31–45 days of daily ATT was similar to the one of those who received 46–60 days of daily ATT.

**Figure 3:**
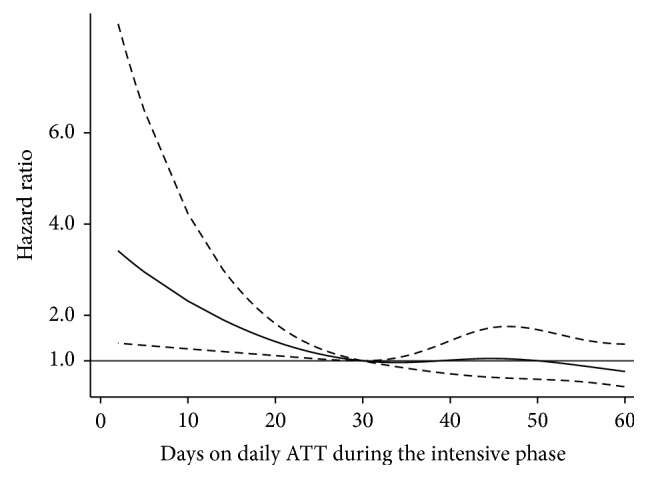
Mortality risk (adjusted hazard ratio and 95% confidence interval) of HIV infected patients with tuberculosis by duration daily treatment during the first two months of antituberculosis therapy (ATT).

**Figure 4:**
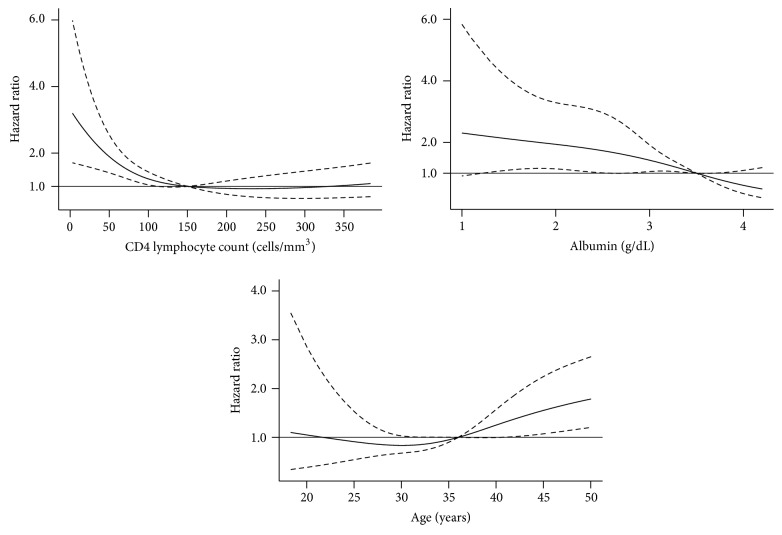
Mortality risk (adjusted hazard ratio and 95% confidence intervals) of HIV infected patients after two months of antituberculosis therapy by CD4 lymphocyte counts, serum albumin, and age.

Multivariable analysis of factors associated with mortality is presented in Table 1 (categorical variables) and Figures 3 and 4 (restricted cubic splines of continuous variables). Patients who started ART within two months of ATT had lower risk of mortality.  Figure 3 describes the adjusted hazard ratio for mortality by duration of daily ATT before switching to intermittent therapy. The mortality risk was inversely proportional to the duration of daily ATT. However, after 30 days of daily ATT, prolonging daily ATT did not significantly reduce the risk of death. Adjusted hazard ratios for mortality by CD4 lymphocyte counts, serum albumin, and age are presented in Figure 4. Patients with low CD4 lymphocyte counts had a higher risk of death but only in those with <150 cells/mm^3^. Patients with lower serum albumin concentrations and elders had also an increased risk of mortality.

## 4. Discussion

In this study, the use of restricted cubic splines allowed for a flexible estimation of the mortality risk by the duration of daily ATT. We found that, in a resource-limited setting with free ATT and ART, the duration of daily ATT before switching to intermittent ATT was inversely correlated with mortality during the first 30 days of treatment. After 30 days, differences were not statistically significant.

Intermittent ATT regimens were endorsed by the WHO based on studies performed in HIV negative patients more than 25 years ago [4] but their efficacy has not been evaluated in randomized clinical trials with HIV infected patients. What is applicable for HIV negative patients might not be true for HIV infected patients. HIV infected patients have higher risk of death, relapse, and acquired drug resistance [5]. Due to immunosuppression, HIV patients might be more dependent on the efficacy of ATT than HIV negative patients, where the immune system is able to support antituberculosis drugs in the fight against tuberculosis bacilli [17]. The mortality reduction observed in our study could be explained by the fact that a more intensified ATT during the first 30 days of treatment might have led to a faster reduction of the mycobacterial burden [18]. These data support the hypothesis that an intensified treatment during the first days of ATT could result in improved survival in HIV patients with tuberculosis [12, 19].

The World Health Organization (WHO) strongly recommends daily ATT during the intensive phase for HIV-associated tuberculosis [20]. The results of this study indicate that prolonging daily ATT longer than 30 days might not have a positive impact on survival, but we did not assess other important outcomes such as failure, relapse, or acquired drug resistance [21]. Therefore, we do not suggest limiting the duration of daily ATT during the intensive phase of ATT to 30 days based solely on the results of this study. In fact, randomized clinical trials have demonstrated that intermittent ATT is associated with higher risk of treatment failure and acquired rifampicin resistance [22, 23], especially in settings with high prevalence of isoniazid resistance such as India [24, 25]. In a recent study of patients enrolled in clinical trials at the National Institute for Research in Tuberculosis in India, HIV infected patients treated with intermittent ATT had a higher risk of acquired rifampicin resistance compared with HIV negative patients [26]. The risk of acquired rifampicin resistance was particularly high among those who were not on ART, which is actually the group at highest risk of developing tuberculosis [26–28].

The study has some limitations. This is an observational study and, unlike clinical trials, the duration of daily ATT before switching to thrice-weekly ATT was not randomly allocated. The study could be biased due to unknown confounders that could have increased the risk of mortality in those patients with shorter duration of daily ATT. New studies are needed to confirm our findings.

## 5. Conclusions 

The results of this study suggest that daily ATT should be given for at least 30 days before switching to thrice-weekly ATT in order to reduce the mortality in HIV infected patients with tuberculosis.

## Figures and Tables

**Table 1:** Baseline characteristics and multivariable analysis of factors associated with mortality in 520 HIV infected patients with tuberculosis who completed two months of antituberculosis therapy in Anantapur, India.

	Baseline characteristics	Mortality risk
	*N* (%)	aHR (95% CI)
Female	184 (35.4)	0.80 (0.54–1.20)
Homeless	15 (2.9)	1.98 (0.77–5.09)
Illiteracy	293 (56.3)	1.40 (0.94–2.09)
Sputum smear positive	110 (21.2)	1.29 (0.87–1.91)
Disseminated TB	27 (5.2)	1.80 (0.94–3.45)
ART initiation		
Before ATT	137 (26.3)	0.81 (0.53–1.24)
Within 2 months of ATT	229 (44)	0.37 (0.23–0.58)
Not initiated	154 (29.6)	1 (reference)
Age (years)	35.5 (30–42)^*^	Figure 4
CD4 count (cells/mm^3^)	136 (71–242)^*^	Figure 4
Serum albumin (g/dL)	3 (2.5–3.5)^*^	Figure 4
Daily ATT (days)	36 (31–48)^*^	Figure 3

^*^Median (interquartile range). ART: antiretroviral therapy; ATT: antituberculosis therapy; aHR: adjusted hazard ratio; CI: confidence interval; TB: tuberculosis. Continuous variables (age, CD4 lymphocyte counts, serum albumin, and number of days on daily ATT) were modeled using restricted cubic splines and their adjusted hazard ratios are presented graphically in Figures 3 and 4.
